# The effect of a new formaldehyde-free binder on the dissolution rate of glass wool fibre in physiological saline solution

**DOI:** 10.1186/1743-8977-10-13

**Published:** 2013-04-12

**Authors:** Russell M Potter, Nassreen Olang

**Affiliations:** 1 Owens Corning Science and Technology Center, 2790 Columbus Road, Route 16, Granville, Ohio 43023-1200, USA

**Keywords:** Binder, Biopersistence, Biosolubility, Dissolution, Fibre, Glass, Wool

## Abstract

The in-vitro dissolution rate of fibres is a good predictor of the in-vivo behavior and potential health effects of inhaled fibres. This study examines the effect of a new formaldehyde-free carbohydrate-polycarboxylic acid binder on the in-vitro dissolution rate of biosoluble glass fibres. Dissolution rate measurements in pH 7.4 physiological saline solution show that the presence of the binder on wool insulation glass fibres has no effect on their dissolution. There is no measurable difference between the dissolution rates of continuous draw fibres before and after binder was applied by dipping. Nor is there a measurable difference between the dissolution rates of a production glass wool sample with binder and that same sample after removal of the binder by low-temperature ashing. Morphological examination shows that swelling of the binder in the solution is at least partially responsible for the development of open channels around the glass-binder interface early in the dissolution. These channels allow fluid to reach the entire glass surface under the binder coating. There is no evidence of any delay in the dissolution rate as a result of the binder coating.

## Background

The critical role of dissolution on the potential health effects of inhaled fibres is well established [1]. Over the last several decades there have been numerous publications on the relation between various physical characteristics of a synthetic vitreous fibre and its dissolution rate in physiological saline solution. These include the chemical composition of the fibre [1], fibre density changes as a result of annealing [2], fibre diameter [3], and the physical changes resulting from the fiberization method [3-5].

In addition to glass fibres, commercial insulation glass wool products typically contain an organic binder to give the strength and mechanical integrity required for their end use application. Although Bauer [5] notes that it is unlikely that fibres with thick binder coatings could reach the deep lung due to aerodynamic considerations, binder is typically found on some fibres during routine microscopic examination of airborne respirable fibres [unpublished observations, M. Kalinowski, Owens Corning Science and Technology Center]. It is therefore desirable to know if such binders provide any protection in the lung environment that would slow the dissolution rate of an otherwise biosoluble fibre.

Glass wool insulation binders, traditionally phenol-formaldehyde based resins, have received some previous study. Mattson [2] measured the dissolution rate in simulated lung fluid of a production glass wool with no applied binder, with phenol-formaldehyde binder, and with that binder removed by low temperature ashing. She found the dissolution rates for the three samples to be the same within the uncertainty of the measurements. Bauer [5] also found that phenol-formaldehyde binder has no effect on the dissolution rate. In addition, his microscopic examination of partially-dissolved samples showed that fluid attack of the resin-glass bond left the fibre surface exposed directly to the fluid early in the dissolution process. Similar experiments with coatings of silicone oil and a silane coupling agent alone, materials commonly used in commercial glass wool insulation binders, showed a similar delamination of the resin-glass interface but at a later point in the dissolution process. Although the silicone-silane coating caused an initial slowing of the dissolution, this did not last long enough to affect significantly the dissolution rate.

The studies just cited show that the phenol-formaldehyde binder traditionally used in commercial glass wool insulation has no effect on the dissolution of the glass fibres in physiological saline solution. In addition, these studies indicated that the absence of a protective effect was due, at least in part, to early attack of the glass-binder interface, which allows access of the fluid to the fibre surface even under thick binder droplets.

More recently, a number of new, formaldehyde-free binders, typically based on carbohydrate-polycarboxylic acid chemistry, have been developed and used in commercial insulation glass wool production. The present study addresses two questions about the effect of such new binders on the dissolution rate of wool insulation glass fibre in physiological saline solution: 1) does a visible layer of the binder affect the dissolution rate of the fibre underneath it and 2) does binder application influence the dissolution rate of fibre not visibly coated with it.

## Results

### Continuous fibre experiment

**Figure 1:**
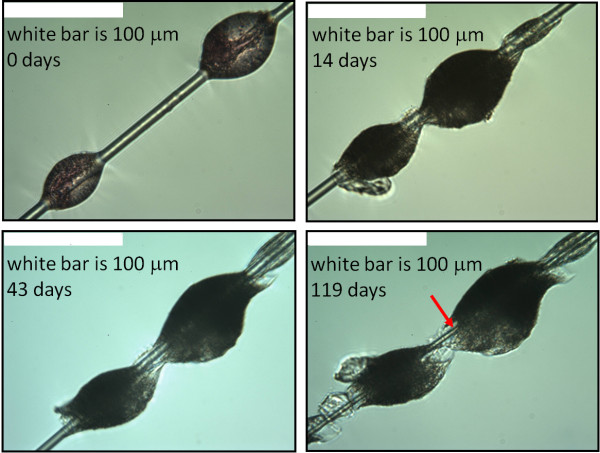
**Large binder droplets at different times in the dissolution process. **Optical micrographs of two large binder droplets from sample 2 at four different times in the dissolution process showing changes in morphology as the dissolution proceeds. The red arrow marks a typical measurement location for fibres in large binder droplets. All micrographs are at the same magnification.

We conducted a first set of experiments on continuous draw glass fibre samples (glass A, Table 1) because the diameter uniformity eliminates any error associated with change in the initial diameter of a fibre along its length. Sample 1 (Table 2) is as-formed fibre with no binder coating; sample 2 (Table 2) was dip-coated with binder. Figure 1 shows a single fibre of sample 2 with two large binder droplets at four times in the dissolution process. The figure illustrates three features which we have found to be characteristic of the dissolution process of fibres with applied binder: 1) early in the dissolution the binder droplets have moved along the fibre relative to each other, 2) at any time period, all parts of the fibre that are visible have decreased in diameter by the same amount, 3) with time the binder droplets increase in size.

**Table 1:** Glass fibre chemical compositions expressed as oxide weight percent

**Oxide**	**Glass A**	**Glass B**
SiO_2_	65.4	67.9
Al_2_O_3_	2.1	1.3
CaO	8.9	7.8
MgO	1.2	2.2
B_2_O_3_	6.6	5.5
MnO	0.9	0.8
Na_2_O	13.2	13.2
K_2_O	0.9	0.6
Fe_2_O_3_	0.4	0.3
total	99.6	99.6

**Table 2:** Samples for dissolution rate measurement

**Sample**	**Glass**	**Binder**	**Dissolution rate in ng/cm**^**2**^**/hr**
1	A	none	271 ± 10
2	A	3x dipped in binder; dried 30 minutes at 100 C; cured 3 minutes at 204 C	263 ± 10
3	B	low temperature ashed to remove production binder	319 ± 22
4	B	production binder	326 ± 9

**Figure 2:**
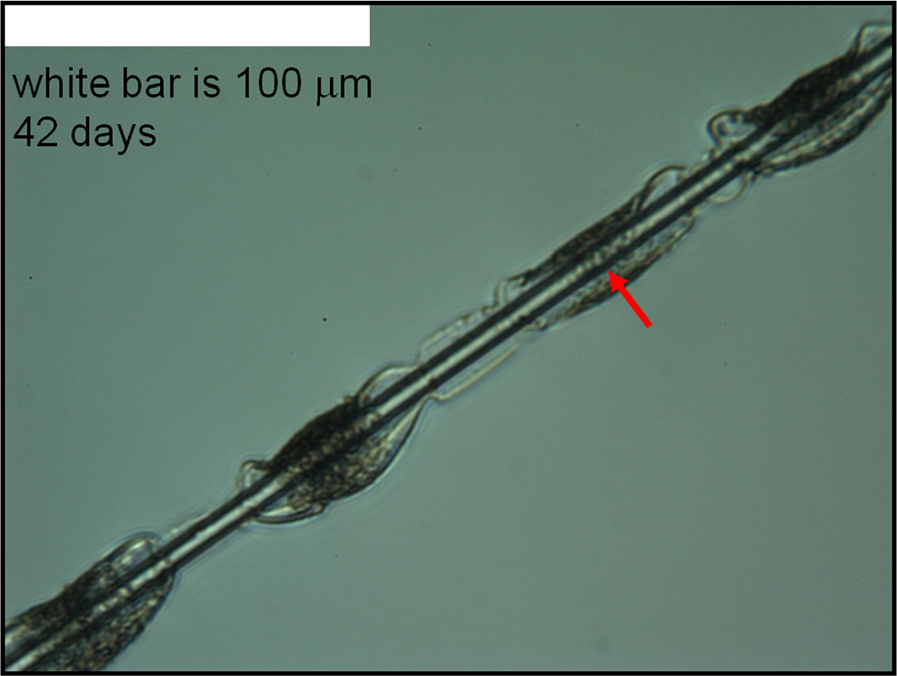
**Small binder droplets with fibre diameter measurable deep within the droplet.** Optical micrograph of four small binder droplets from sample 2 in which it is possible to measure the fibre diameter at any point in the droplet. The red arrow marks a typical measurement location for fibres in small binder droplets.

Throughout the dissolution, we measured the fibres as far into the binder droplets as possible wherever they happened to be on the fibre, as shown, for example, by the red arrows in Figures 1 and 2. The position of the binder droplets on the fibres had generally stabilized after several weeks.

**Figure 3:**
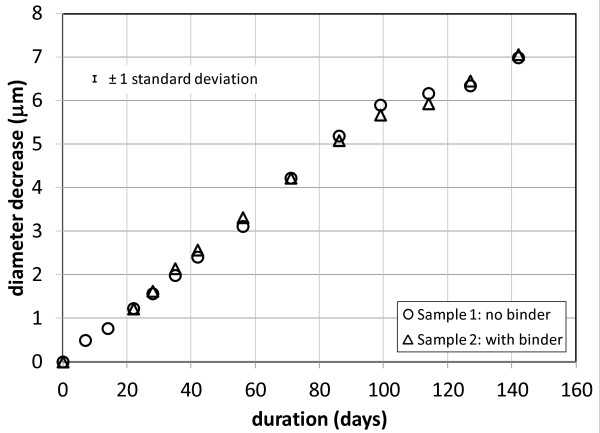
**Average continuous draw glass fibre diameter decrease with time. **The average fibre diameter decrease with dissolution time from measurements on eight individual fibres for continuous draw glass fibre samples 1 and 2. The indicated error is typical of a set of measurements on 8 individual fibers.

Figure 3 shows the average measured diameter decrease for samples 1 and 2 from an initial diameter near 9.5 μm to the point at which the fibre diameters became too small to measure. The two samples were measured at the same time using physiological fluid from the same reservoir. Periodic measurement of fluid pH during this time showed it to be 7.38 ± 0.08. The dissolution rates calculated from the data are in Table 2.

### Production wool experiment

**Figure 4:**
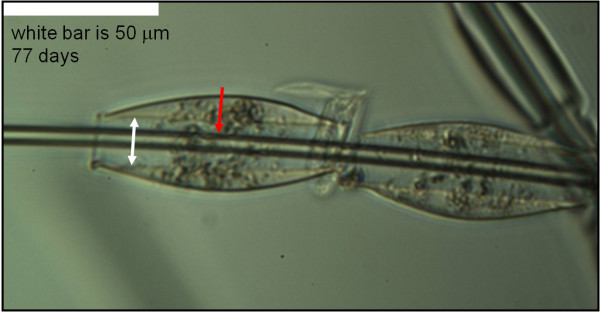
**An open channel between binder and fibre. **Optical micrograph of two small binder droplets from sample 4 showing the open channel around the fibres (white arrow). The red arrow marks a typical measurement location for fibres in small binder droplets.

In this study we measured the dissolution rates of production glass wool fibres (glass B in Table 1). Sample 4 (Table 2) is as-formed fiber with the production binder present. Sample 3 (Table 2) is the same material after exposure to low-temperature oxygen plasma, which removes all organic matter without altering the glass fibre. Because smaller binder droplet size allows us to measure fibre diameter more deeply within the droplet, we choose measurement locations on the fibre with smaller binder droplets such as in Figure 4. The binder droplets generally remained throughout the dissolution, but, as in the first experiment, some droplets moved along the fibres in the initial several weeks of the dissolution process. Throughout the dissolution, we measured the fibres as far into the binder droplets as possible wherever they happened to be on the fibre as shown, for example, by the red arrow in Figure 4. In those cases in which the binder had disappeared, we continued to measure the diameter of the fibre at the location where we had last seen the binder. Figure 4 shows clearly a morphology that generally develops in the binder as the dissolution proceeds but which the opacity of the binder droplets obscures somewhat in the other figures – an open channel along the fibre (white arrow in Figure 4). These channels typically develop within the first several weeks of the dissolution process and have a diameter significantly greater than the initial diameter of the fibre they enclose.

**Figure 5:**
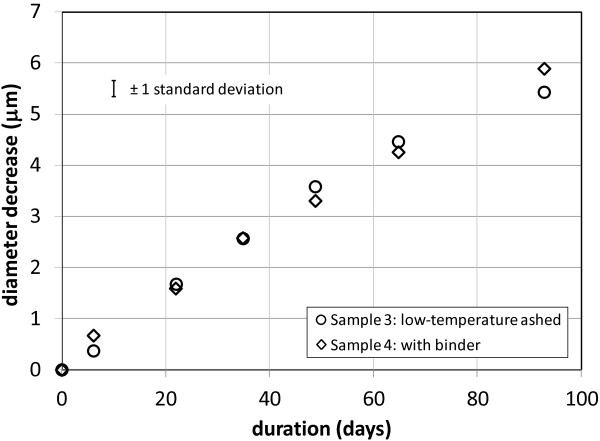
**Average glass wool fibre diameter decrease with time. **The average fibre diameter decrease with dissolution time from measurements on eight individual fibres for production glass wool samples 3 and 4. The indicated error is typical of a set of measurements on 8 individual fibers.

Figure 5 shows the average measured diameter decrease for the production wool samples 3 and 4 until about half of the fibre diameters were too small to measure. The initial measured fibre diameters range from ~4 μm to ~13 μm and are typically ~9 μm. This is significantly larger than the average fibre diameter of the production wool samples, which is in the range 5–6 μm. We chose the larger diameter fibres for ease of measurement and to allow the dissolution to be followed for a longer time. Examination of the diameter change with time for individual fibres shows that a fibre’s initial diameter has no influence on its dissolution rate. The two samples were measured at the same time using physiological fluid from the same reservoir. Periodic measurement of solution pH during this time showed it to be 7.33 ± 0.08. The dissolution rates calculated from the data are in Table 2.

## Discussion

### Fibre dissolution under binder droplets

The data in Figures 3 and 5 and the dissolution rate constants in Table 2 calculated from them show no measurable difference between the dissolution of fibre within a binder droplet and fibre with no binder. The mobility of binder droplets along the fibres shortly after the dissolution process has begun indicates that any pre-existing bond between binder and fibre is soon broken, giving the dissolution fluid access to the fibre surface. If the binder droplets slowed the dissolution to a measurable extent before this occurred, we would expect to see later in the dissolution locally thick areas along the fibre. These would be particularly evident in the first experiment with the uniform diameter, continuous draw fibres. We found no such areas in either set of experiments.

**Figure 6:**
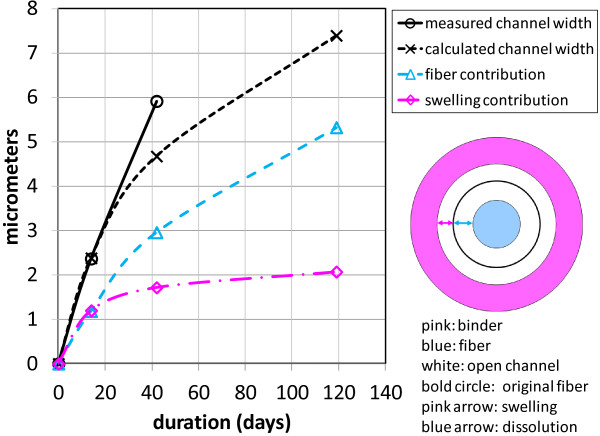
**Open channel width change with time. **Width of the open channel as a function of dissolution time from direct measurement and as calculated from swelling of the binder and dissolution of the fibre. The diagram at the right is a cross-section perpendicular to the fibre at the position of the white arrow in Figure 4 (77 hours into the dissolution).

Analysis of the morphology of binder and glass during the dissolution process indicates that swelling of the binder due to water absorption is a significant factor in allowing access of the dissolution fluid to fibre surfaces enclosed in binder. During the dissolution process, water absorption by the binder causes it to swell. This swelling will tend to break any bonds at the fibre-binder interface and to promote the formation of an open channel around the fibre. At the same time, dissolution of the fibre will decrease its diameter, further enlarging this channel. We calculated the amount of swelling from the diameter change of binder droplets such as in Figure 1. We measured the increase in the outer diameter of the channel and the decrease in the fibre diameter from micrographs such as Figure 4. The results are in Figure 6. For the first several weeks the swelling and the fibre dissolution make approximately equal contributions to the open channel width. Later, the fibre dissolution becomes the major contributor, though the calculation predicts a narrower open channel that we measured. The diagram at the right in Figure 6 shows the contribution of binder swelling and fibre dissolution to the open channel at a point corresponding to the white arrow in Figure 4 (after 77 days of dissolution).

### Fibre dissolution with no visible binder

It is possible that some component of the binder forms a protective coating in the fibre that is too thin to see under microscopic examination as Bauer found for a silicone-silane coating [5]. We found no evidence for this in either set of experiments. Both the continuous draw and the production wool fibres decreased in diameter regardless of droplet location at a rate equal to that of fibres without binder.

## Methods

### Sample preparation

Glass A is a production wool glass that was formed into continuous fibre of uniform 9.5 μm diameter using a laboratory continuous draw process. Sample 1 in Table 2 is this fibre with no further treatment. We coated individual fibres with binder by dipping them into a binder solution three successive times with a brief drying at 100 C between each dip. Finally we dried the fibres for 30 minutes at 100 C and cured them for 3 minutes at 204 C. This resulted in fibres with small droplets of cured binder along their lengths at irregular intervals with no obvious coating in between. Sample 2 in Table 2 is this fibre. Glass B is production glass wool insulation which already contained binder. Sample 4 in Table 2 is this fibre with no further treatment. Sample 3 is this fibre treated with low-temperature oxygen plasma to remove all organic material. The binder on samples 2 and 4 is a carbohydrate-polycarboxylic acid based formulation of the type described by Hawkins [6].

### Dissolution rate measurement

We determined fibre dissolution rate by direct measurement of fibre diameter using an optical microscope as documented elsewhere [3]. In this method, individual fibres are glued onto a plastic mount so that they span an open slot in the mount. This mount fits into a closed, transparent, flow-through cell which directs a constant flow of the physiological fluid past the fibres. Immersion in a constant temperature bath maintains the cell at 37 C. Periodically the cell is removed from the bath and the diameters of individual fibres measured. A linear fit of diameter decrease with time yields the dissolution rate constant, k_dis_, in units of ng/cm^2^/hr with an uncertainty which is the standard error of the fit.

In the work reported here, solution flow rate was 0.1 ml/min. Due to the small fiber surface area exposed to the fluid, this is well within the region in which fiber dissolution rate is independent of solution flow rate [3]. For each fiber sample we measured the diameters of eight individual fibres approximately every two weeks. The edge of the mount slot, the distinct morphologies of the individual fibres, and a vernier on the microscope stage allowed us to identify the measurement location on each fibre. The physiological fluid was a modification of Gambles solution at a pH near 7.4 as described by Mattson [7]. It was not always possible to see the fibre through a binder droplet clearly enough to measure its diameter deep within the droplet (Figure 1). But it is generally possible to do this with smaller droplets after several weeks in the physiological fluid (Figures 2 and 4).

## Conclusions

Under the conditions of these experiments we found no evidence of any impact of the carbohydrate-carboxylic acid binder on the measured in-vitro dissolution rate of the two biosoluble insulation glass wool compositions.

## Competing interests

Both authors are employed by Owens Corning. This research was performed at the Owens Corning Science and Technology Center on materials manufactured by Owens Corning.

## Authors’ contributions

Both authors designed the experiments and participated in fibre sample preparation. RP measured the dissolution rates of the samples and drafted the manuscript. Both authors read and approved the final manuscript.
